# The changing neglected tropical disease landscape in Africa: implications for policy, practice, and strengthening health systems

**DOI:** 10.1093/haschl/qxaf136

**Published:** 2025-07-31

**Authors:** Philip Downs, Andrew Tate, Danny Harvey, Margaret C Baker

**Affiliations:** Sightsavers, Haywards Health, West Sussex RH16 3BW, UK; Sightsavers, Haywards Health, West Sussex RH16 3BW, UK; Sightsavers, Haywards Health, West Sussex RH16 3BW, UK; Department of Global Health, Georgetown University, Washington, DC 20057, United States

**Keywords:** universal health coverage, mainstreaming, integration, primary health care, strengthening health systems, neglected tropical diseases (NTDs), mass drug administration

## Abstract

**Introduction:**

Mass drug administration (MDA) has reached up to 1 billion people annually to combat neglected tropical diseases (NTDs). This study explores the evolving NTD landscape in Africa amid shifting disease patterns, funding cuts, and universal health coverage goals.

**Methods:**

We analyzed data from ESPEN and Sightsavers databases to assess historical trends and forecast future treatment needs.

**Results:**

By 2027, few or no MDAs for lymphatic filariasis and trachoma will be required and MDAs for onchocerciasis dropped sharply after 2024. In contrast, schistosomiasis and soil-transmitted helminths continue to require widespread MDA.

**Conclusion:**

To secure elimination gains, remaining MDAs must be completed and broader health systems approaches adopted.

## Introduction

Universal health coverage (UHC) remains a defining goal for global health and a pressing priority for African health systems, where more than 600 million people still lack access to essential services.^1^ Achieving UHC requires expanding access to care, strengthening primary health systems, and reaching marginalized populations—challenging goals given workforce shortages, financing gaps, and low domestic financing coupled with low prioritization by external funders of health systems.^1^

Neglected tropical disease (NTD) programs in Africa offer timely lessons of what can be achieved through coordinated action—even in the most resource-constrained settings. They also offer the opportunity to reflect on the future of disease-control programs following recent major reductions in donor funding, including for vertically delivered, disease-specific, interventions.^2,3^

Neglected tropical diseases are a group of 21 preventable and treatable diseases that disproportionately affect impoverished, underserved communities, causing significant health, social, and economic harm.^4,5^ These include lymphatic filariasis, which can lead to severe limb swelling (elephantiasis); trachoma and onchocerciasis, which are the 2 leading infectious causes of blindness; and schistosomiasis and soil-transmitted helminthiasis, which particularly impact children and pregnant women, contributing to anemia, malnutrition, and impaired cognitive development.^1^

Over the past 2 decades, campaign-style, mass drug administrations (MDAs) have been used to prevent and treat these 5 diseases.^6,7^ Mass drug administrations involve the periodic, population-wide distribution of safe medications—regardless of individual infection status—in areas where disease prevalence exceeds defined thresholds. Highly effective at reducing disease burden at scale, these can be delivered at the low cost of approximately 50 cents per person treated, with demonstrated economic returns on investment.^8,9^ Using these approaches, up to 1 billion of the world's most marginalized people have been reached annually,^10^ with evidence that women and girls are reached equally as well as men.^11^ Mass drug administrations effectively reduce the risk of infection to very low rates, after which they can be stopped. As a result, the number of people requiring NTD treatment has decreased by 26% from 2.19 billion in 2010 to 1.62 billion in 2022 and, as of December 2023, at least 1 NTD has been eliminated in 50 countries.^12^

The World Health Organization (WHO) 2021–2030 NTD Roadmap calls for a paradigm shift in addressing NTDs: transitioning from vertical programs to integrated services embedded within routine health systems and shifting from donor-driven agendas to nationally led health services.^4^

This report examines the rapidly evolving landscape of NTDs in Africa, highlighting the urgency for policy and practice adaptation—especially in the context of UHC and shifting global health funding.

## Data and methods

We used publicly accessible data from the ESPEN (Expanded Special Project for Elimination of Neglected Tropical Diseases) website,^13^ which curates district-level data reported to WHO by Ministries of Health (MoH) in the WHO Africa Region. Treatment forecasts were generated assuming that future MDA rounds will be delivered as scheduled, and that prevalence will reduce following an expected trajectory, assuming 100% survey success. While complete success is unlikely, it is expected to be close. For example, in a study by Burgert-Brucker et al,^14^ 87% of implementing units met lymphatic filariasis program goals within the expected time frame.

ESPEN program data were downloaded using an Application Programming Interface connection.^15^ To address the inherent lag in available data, forecasts were updated based on the latest MoH submissions on the ESPEN website (where available) and using Sightsavers’ internal program-monitoring reports. Sightsavers is a global nongovernment organization (NGO) that has worked with governments across 34 countries to provide financial, operational, and technical support to NTD programs. Projects were made based on historic trajectory and do not account for the, as yet unknown, impact of global funding cuts.

Where the status of onchocerciasis is unknown at district levels, we factored in 10–15 additional years of presumptive MDAs starting in 2027. This represents a “worst case” scenario and is reflected in the increase in treatments forecasted in 2027. In the case of schistosomiasis, the count of implementation units (IUs) (normally, districts) represents all those considered endemic; however, not all IUs will be targeted for treatment on an annual basis, so this is a conservative estimate.

Consolidated results were fed into the business intelligence tool PowerBI to facilitate analysis.

## Results

**Figure 1.**
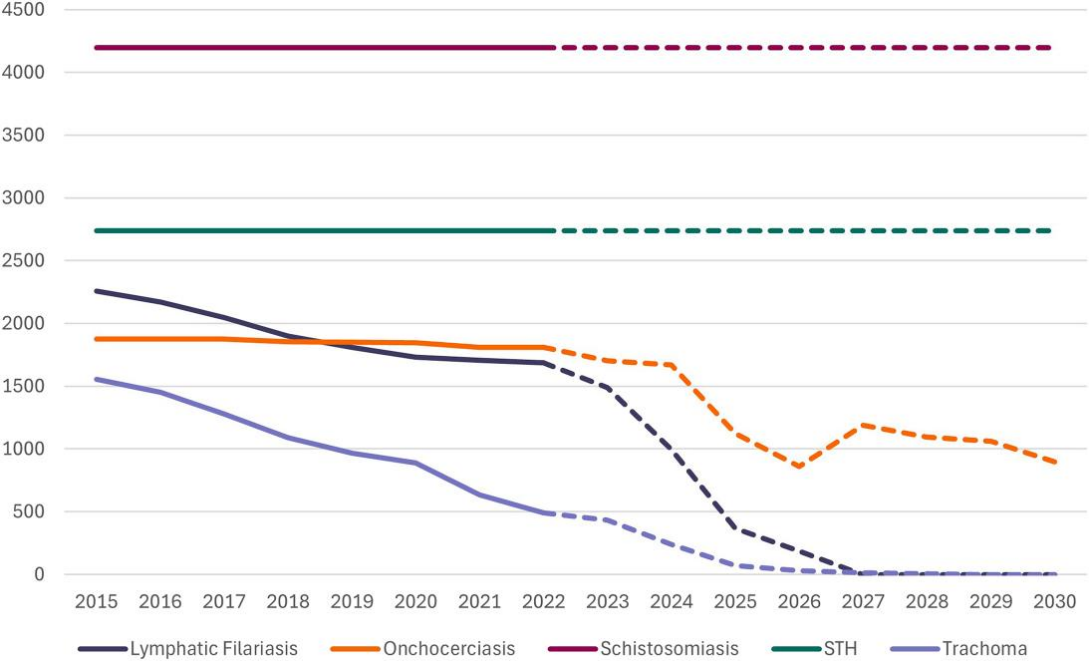
Number of implementation units in the WHO Africa Region requiring mass drug administration by year and disease. Data source: Publicly accessible data from the ESPEN website, which curates district-level data reported to WHO by Ministries of Health in the WHO Africa Region. Counts up to year 2022 are based on actual data (solid lines), after which counts are based on forecasts (dashed lines).

Abbreviations: ESPEN, Expanded Special Project for Elimination of Neglected Tropical Diseases; STH, soil-transmitted helminthiasis; WHO, World Health Organization.

Figure 1 shows the number of IUs (typically districts) where MDA interventions have been implemented up until 2022 and projected thereafter. Clear temporal differences are observed among diseases. Treatments for lymphatic filariasis and trachoma have been scaling down since before 2015 and, based on these projections, few or no MDAs will be needed by 2027. Treatment needs for onchocerciasis remained more or less constant up until 2022, with a more rapid drop-off projected after 2024 as more areas reach the threshold required to stop drug treatment. By contrast, requirements for population-level treatment for soil-transmitted helminths and schistosomiasis are projected to remain high for the foreseeable future, with 41 countries requiring MDA for schistosomiasis and 38 for soil-transmitted helminthiasis in 2023. We do not factor into the equation the need for the treatment of younger children for schistosomiasis, which is dependent on a pediatric praziquantel formulation, which is not donated.

## Discussion

Our analysis highlights a rapidly changing NTD control landscape in Africa. First, great progress has been made toward eliminating lymphatic filariasis, trachoma, and onchocerciasis—with relatively few MDAs required going forward. However, although many countries have already achieved very low levels of infection, recent large-scale funding cuts have introduced significant uncertainty. Without relatively small, but critical investments, hard-won gains could now be at risk.

To fully understand the implications of this progress, it is crucial to distinguish between disease elimination and eradication. Eradication, defined as zero global incidence of a specific pathogen,^16^ has only been achieved to date for smallpox, while other diseases are close (eg, polio and guinea worm). In elimination, disease and/or infection is reduced to very low levels, large-scale control activities are no longer needed, the majority of people are no longer at risk, and the risk of recrudescence is very low. However, ongoing surveillance and response to localized emergence of new cases, such as those reported for lymphatic filariasis in Sri Lanka^17^ and Egypt,^18^ are still required. Additionally, these diseases are chronic and patients often require treatment for years after the infection has gone. Continuation of MDAs for longer than planned should also be anticipated in a small number of districts. For example, estimated time to elimination of lymphatic filariasis in Sierra Leone, Nigeria, Tanzania, and Ghana was extended due to factors including an Ebola outbreak, high baseline prevalence, or proximity to endemic communities along national borders.^19-22^ Similarly, small pockets of trachoma have persisted in Ethiopia, where very high rates of recrudescence have been observed.^23^ This requires strong surveillance systems and empowered local-level capacity to respond to signals of new or ongoing infection.

Second, our analysis shows that the need for deworming for schistosomiasis and soil-transmitted helminthiasis is projected to continue at-scale for the foreseeable future. In many countries, deworming efforts have been co-delivered alongside MDAs for lymphatic filariasis, trachoma, and onchocerciasis—the need for which is rapidly decreasing. Additionally, recent publications on deworming note the need to often expand treatment beyond school-age children to include adults and pre–school-aged children,^24^ while also making the case for more targeted deworming—moving away from district-level treatment to more local geographies.^25^

New delivery modalities for deworming therefore need to be identified. School health programs have long been a platform for deworming programs, which can be delivered with other health interventions.^26^ For example, Sightsavers partnered with the Ministries of Health and Education in Liberia to integrate deworming and eye health in schools. Initial targets for vision screening and training of eye health and deworming agents were exceeded. Similarly, deworming has been integrated with nutrition in schools, with the scale-up of national school-meal programs in parallel with deworming programs.^27^ However, school-based approaches are limited in their ability to reach children and youth who are outside the educational system, pre–school-aged children, and women of childbearing age.^28^ Newer test-and-treat deworming strategies, particularly in areas of low prevalence and intensity of infection, could alternatively be delivered through primary health care. Health workers would use low-cost screening protocols (based on clinical symptoms and exposure) or point-of-care diagnostic tests.^29^ This will require investment in broader health systems, including building the capacity of health center and local laboratory staff, adapting national drug procurement, and developing treatment guidelines.

This shift in the NTD landscape requires all stakeholders (including governments at their different levels, donors, and NGOs) to reflect on their roles going forward. They each have an important role to play, but it is likely to look quite different. For example, Sightsavers is an NGO that historically has supported national NTD programs to capacitate over 100 000 volunteer health workers and 36 000 teachers, supporting the distribution of over 1.7 billion NTD treatments to date. As well as continuing to contribute to reduce preventable causes of blindness and NTD-caused disabilities, Sightsavers is committed to broaden its support to strengthen systems and policies that accelerate disease elimination and reduce the impact these diseases have on the poor. This includes strengthening disease-surveillance and response mechanisms, promotion of personal preventive measures, and the integrated management of long-term NTD morbidity and psychosocial support within the health system. This inevitably will mean leveraging and adapting community- and school-based platforms to deliver health services to some of the most marginalized populations globally.

Beyond their disease-specific goals, NTD programs also offer a blueprint for designing and delivering public health services that reach marginalized populations at scale. Lessons from MDA campaigns—such as adapting delivery strategies to local contexts, developing strong partnerships with pharmaceutical companies, and coordinating across sectors such as health and education—are highly applicable to other health priorities. This includes leveraging trusted community agents such as volunteer drug distributors. This trained informal workforce, also often engaged in other health activities, provided manpower where the health system lacked it; reached the community through known and trusted “drug distributors”; and by going to the community, effectively reached vulnerable populations.^30^ They have been essential to the success of NTD programs and now offer a valuable resource that could be harnessed by broader health systems to expand the reach of other health interventions. This would address some of the barriers to UHC in Africa identified, especially the shortage of trained health workers and inequitable access to services—including for women, girls, and vulnerable populations.

The data presented here highlight a pivotal moment for 5 NTDs in Africa—one that coincides with a significant reduction in external funding and an urgent need to accelerate progress toward UHC. For policymakers focused on strengthening health systems, this moment presents an opportunity to leverage the infrastructure, workforce capacity, and community engagement built over decades of programs targeting NTDs and other diseases. At the same time, those shaping the future of disease control have a chance to reimagine how programmatic activities can better align with national health systems, while also recognizing the continued need for dedicated funding to sustain hard-won gains and prevent resurgence.

## Supplementary Material

qxaf136_Supplementary_Data
